# An IL-4/21 Inverted Cytokine Receptor Improving CAR-T Cell Potency in Immunosuppressive Solid-Tumor Microenvironment

**DOI:** 10.3389/fimmu.2019.01691

**Published:** 2019-07-19

**Authors:** Yi Wang, Hua Jiang, Hong Luo, Yansha Sun, Bizhi Shi, Ruixin Sun, Zonghai Li

**Affiliations:** ^1^State Key Laboratory of Oncogenes and Related Genes, School of Biomedical Engineering, Renji Hosptial, Shanghai Jiao Tong University, Shanghai, China; ^2^State Key Laboratory of Oncogenes and Related Genes, Renji Hospital, Shanghai Cancer Institute, Shanghai Jiao Tong University School of Medicine, Shanghai, China

**Keywords:** CAR-T cells, inverted cytokine receptors, interleukin-4, interleukin-21, immunosuppressive tumor microenvironment

## Abstract

Incorporation of inverted cytokine receptor (ICR) such as interleukin (IL)-4 vs. IL-7 (4/7) ICR is one strategy to improve the antitumor activities of chimeric antigen receptor (CAR) modified T (CAR-T) cells facing immunosuppressive cytokines. Here we report a novel interleukin (IL)-4 vs. IL-21 ICR (4/21 ICR) that enhanced CAR-T cell potency in IL-4^+^ tumor milieu via a different working-mechanism from 4/7 ICR. Upon IL-4 stimulation, 4/21 ICR activated the STAT3 pathway and promoted Th17-like polarization and tumor-targeted cytotoxicity in CAR-T cells *in vitro*. Furthermore, 4/21 ICR-CAR T cells persisted and eradicated established IL-4^+^ tumors *in vivo*. Thus, 4/21 ICR is a promising clinical CAR-T cell therapeutics for solid tumors rich in IL-4.

## Introduction

Although the adoptive transfer of T cells genetically engineered with a chimeric antigen receptor (CAR) has shown great promise as a therapeutic for hematological malignancies (1, 2), limited success has been made in the application of CAR-T cells for the treatment of solid tumors. Existing theories believe that the immunosuppressive microenvironment of solid tumors, which features limited nutrients and oxygen, accumulation of inhibitory cells and cytokines, vascular disturbances, and endothelial dysfunction, poses a major obstacle for cancer immunotherapy including CAR-T cell therapy (3, 4).

To increase T cell potency in the suppressive microenvironment, CAR-T cells could be modified to become intrinsically resistant to anti-inflammatory cytokines. Notably, an inverted cytokine receptor (ICR), in which the ectodomain of the interleukin (IL)-4 receptor is fused to the endodomain of the IL-7 receptor (4/7 ICR), has been designed to protect CAR-T cells from IL-4 suppression (5, 6). 4/7 ICR could accept immunosuppressive IL-4 but convert the downstream signal to that of the immunostimulatory IL-7 receptor. Upon IL-4 engagement, CAR-T cells armored with 4/7 ICR retained the Th1 phenotype and cell viability *in vitro*, and persisted with powerful antitumor activity *in vivo* (5).

In addition to IL-7, IL-21 has also been reported to promote T cell-mediated tumor rejection. Tumor-directed T cells expanded in IL-21 conditional media showed enhanced antitumor efficacy in a B16-melanoma mouse model (7). Human CAR-T cells engineered to express IL-21, efficiently eliminated tumor cells with long-term persistence in immunodeficient mice (8). Thus, as a γ-chain cytokine like IL-7, IL-21 could be a hopeful candidate to constitute a novel IL-4-related ICR. IL-21 is a pleiotropic cytokine that plays critical roles in modulating the effector functions of CD8^+^ T cells and polarization of naïve CD4^+^ T helper (Th) cells (9). Hence, it is interesting to investigate the different efficacy and working-mechanisms in CAR-T cells between 4/7 ICR and 4/21 ICR.

In the current study, 4/21 ICR-CAR T cells achieved rapid tumor eradication in the presence of IL-4, with a comparable efficiency to that of 4/7 ICR-CAR T cells. Evidences indicated that 4/21 ICR-CAR T cells polarized to the Th17-like phenotype rather than the Th1 phenotype of 4/7 ICR-CAR T cells (5), suggesting a distinct mechanism on promoting antitumor activities between 4/7 ICR and 4/21 ICR.

## Materials and Methods

### Mice

Female 6-week-old NOD.Cg-*Prkdc*^*scid*^Il2*rg*^*tm*1*Sug*^/JicCrl (NOG) mice were introduced by Vital River Co. (Beijing, China) from Central Institute for Experimental Animals (CIEA) of Japan and housed under specific pathogen-free conditions.

### Cell Lines

Huh-7, PLC/PRF/5, and HEK-293T were obtained from the ATCC. SMMC-7721 cells were obtained from the Cell Bank of the Shanghai Institute of Cell Biology, Chinese Academy of Sciences. The HCC cells were engineered to produce IL-4 by transduction with the GFP-F2A-IL-4 lentivirus. For the transduction, 2 × 10^5^ /ml cells were placed in a 24-well plate with lentiviral supernatant. The transgene expression was analyzed by GFP fluorescence using flow cytometry. The IL-4 secretion was further confirmed by ELISA (Supplementary Figure S1). All these cells were cultured in DMEM supplemented with 10% FBS.

### Generation of Lentiviral Vector Encoding CAR and ICR

The nucleotide sequence encoding for the signal peptide and extracellular domain of the IL-4 receptor α was fused to the transmembrane and intracellular domain of IL-7 or IL-21, respectively, to generation 4/7 or 4/21 ICR coding sequences. Then the ICR sequences were linked with the sequence encoding anti-GPC-3-28zCAR by a self-cleaving 2A (F2A) sequence with the restriction sites MluI and SalI. Finally, the CAR-F2A-ICR DNA fragments were integrated into pRRLSIN vector after enzymatic digestion with MluI and SalI.

### Lentivirus Production

The lentiviral supernatants were produced by HEK-293T cells, which were transfected with 5.4 μg of vectors that encoded CAR and ICR, 6.2 μg of Prre, and pREV vector and 2.4 μg of pVSVG vector for envelope expression, by 60 μg of polyethylenimine (PEI, Polysciences, Inc.). The supernatants were collected 48 h after transfection and further concentrated 80-fold by polyethylene glycol 8000 (PEG 8000, Sigma-Aldrich) precipitation at 4°C overnight.

### Transduction and Expansion of Human T Cells

The activation, transduction, and expansion of human T cells have been described in the previous study (10). Briefly, T cells in peripheral blood mononuclear cells (PBMCs) were activated by Dynabeads (ThermoFisher Scientific) for 48 h. Then 1 × 10^6^/ml cells were transduced with lentivirus in a RetroNectin-precoated 24-well plate by centrifugation under 1,800 rpm for 40 min. Human T cells were cultured in AIM-V (Gibco) supplemented with 2% ABS added IL-2 (500 U/ml, Huaxin Biotech., Shanghai, China).

### Flow Cytometry

CAR-T cells treated with IL-4 were harvested and washed with ice-cold phosphate buffered saline (PBS) followed by incubation with antibodies for 30 min on ice. After being washed twice, cells were subjected to analysis by flow cytometry. For plasma samples, Lysing Solution (BD) was added to lyse red blood cells after antibody incubation.

### Western Blot Analysis

CAR-T cells were rested in serum-free AIM-V without cytokines overnight, and subsequently stimulated with IL-4 (20 ng/ml, Peprotech) for 30 min. The cell lysates were denatured for SDS-PAGE and immunoblotted with monoclonal antibodies listed in Supplementary Table S1.

### Quantitative Real-Time Polymerase Chain Reaction (qPCR)

CAR-T cells were treated with IL-4 (20 ng/ml) for 72 h and collected for RNA extraction by TriZol (Life Tech.). The total RNA was further reverse transcribed to cDNA using GoScript Reverse Transcrition System (Promega). The mRNA expression was analyzed by qPCR using SYBR Premix Ex Taq II (TaKaRa) with primers listed in Supplementary Table S2. The relative quantification of targeted genes was calculated with the ΔΔCt method.

### Cytotoxicity Assays *in vitro*

The cytotoxicity assay has been described in the previous study (11). In brief, CAR-T cells pre-treated with IL-4 (20 ng/ml) were co-cultured with target tumor cells for 18 h and the lactate dehydrogenase activity in supernatant was detected by the CytoTox 96 Non-Radioactive Cytotoxicity Assay (Promega).

### Tumor Re-challenging Assays

CAR-T cells were co-cultured with IL-4-producing Huh-7 cells at the effector: target ratio of 1:1 for the first round of stimulation. Tumor clearance was confirmed as no adherent cell appeared in the microscopic field after 48 h. CAR-T cells were then analyzed for the expression of inhibitory receptors or further cultured with IL-4-producing Huh-7 cells at the effector: target ratio of 1:20 for another 48 h. CAR-T cells were collected to re-analyze the expression of inhibitory receptors. Wells were gently rinsed by PBS twice to remove non-adhesive T cells and the tumor cells in wells were stained by crystal violet.

### Tumor Xenograft Models

NOG mice were subcutaneously inoculated with 3 × 10^6^ tumor cells on the right flank. When tumor burden reached the scheduled level (~100 mm^3^ for SMMC-7721 and 200 mm^3^ for PLC/PFR/5), mice were randomly subdivided into four treatment groups and intravenously injected with 3 × 10^6^ CAR-T or untransduced T cells. Tumor dimensions were measured with calipers every 3–4 days. The tumor volumes were calculated by the formula: V = (length × width^2^)/2. The peripheral blood samples were collected for the analysis of T-cell survival on day 14 after T-cell infusion.

### Statistical Analysis

Statistical analysis was performed using GraphPad Prism 5.0 and SPSS 17.0. The Unpaired Student's *t*-test was used to compare the two groups. The One-way ANOVA with Tukey *post-test* was used to determine the statistical significance for three-group comparisons. All experimental data are presented graphically or by mean ± standard deviation (SD).

## Results

### IL-4 Induced a Transformed STAT3 Phosphorylation in 4/21 ICR-CAR T Cells

**Figure 1:**
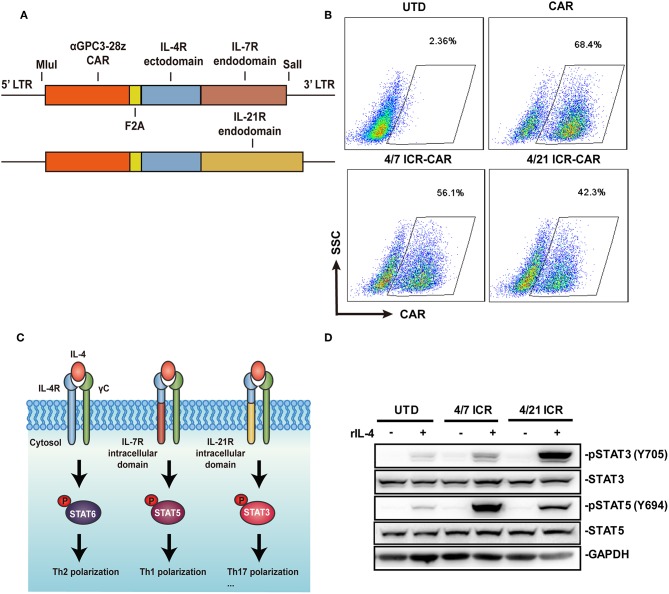
Generation of 4/21 ICR-CAR T cells. **(A)** Schematic representation of 4/7 and 4/21 ICR CARs. **(B)** Flow cytometric analysis of the transgenic efficiency of 4/7 and 4/21 ICR CARs. **(C)** Simplified model for IL-4 signaling pathway through native IL-4 receptors or 4/7 and 4/21 ICR. **(D)** Altered downstream signaling of 4/7 and 4/21 ICR as determined by STAT3/5 phosphorylation using Western blot. Representative results from one of three or more independent experiments are shown.

Similar to the design of 4/7 ICR (5), 4/21 ICR was constructed by fusing the extracellular domain of the IL-4 receptor to the transmembrane and intracellular domain of the IL-21 receptor (Figure 1A). The transduction efficiency of 4/7 ICR CAR and 4/21 ICR CAR is around 50% and relatively lower than that of CAR alone (Figure 1B). Tumor-associated IL-4 can induce Th2 differentiation via STAT6 phosphorylation to directly inhibit T-cell cancer immunity. In our assumption, IL-4 recognition by 4/21 ICR should result in STAT3 phosphorylation, a hallmark of IL-21 signaling, and increase the T cell activities (Figure 1C). As shown in Figure 1D, in the presence of IL-4, STAT3 was strongly phosphorylated in 4/21 ICR-CAR T cells, accompanied with a weak phosphorylation of STAT5, which was reported to transiently occur in IL-21 signaling (12), and as previously reported, increased STAT5 phosphorylation was observed in 4/7 ICR-CAR T cells exposed to IL-4 (5).

### 4/21 ICR-CAR T Cells Demonstrated Th17-Like Phenotypes in the Presence of IL-4

**Figure 2:**
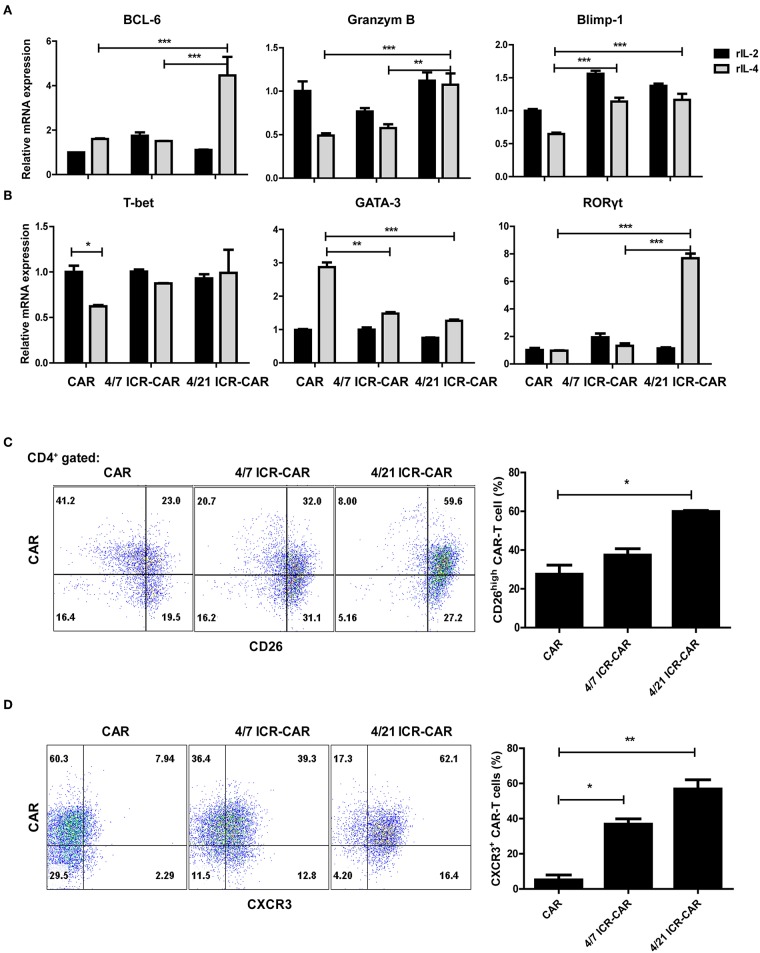
Th17-like polarization of 4/21 ICR-CAR T cells. **(A,B)** Relative mRNA expression of IL-21 target genes and specific transcriptional factors for T helper subsets (T-bet for Th1, GATA3 for Th2, and RORγt for Th17) after IL-4 exposure (20 ng/ml for 48 h) were measured by qPCR. **(C,D)** Flow cytometric analysis of CD26 and CXCR3 expression of 4/7 and 4/21 ICR CARs after IL-4 exposure (20 ng/mL for 48 h). Representative results from one of three independent experiments are shown. *n* = 3 samples for each group; Graphic results are presented as mean ± SD; *p < 0.05; **p < 0.01; ***p < 0.001, one-way ANOVA with Tukey *post-test* for multiple comparison.

We next measured the mRNA expression of IL-21 target genes in T cells after IL-4 exposure. The expression of Bcl-6, a transcriptional regulator that maintains memory cell properties (13), was significantly increased in 4/21 ICR-CAR T cells, while the expression of Blimp-1, a transcriptional repressor associated with effector functions and memory responses (14), was reserved. In addition, the elevated expression level of Granzyme B was also observed (Figure 2A). These results indicate that 4/21 ICR-CAR T cells might sustain memory T cell homeostasis with enhanced effector functions, which is not surprising in light of the multifaceted roles of IL-21 in T cell differentiation (9).

Unlike the IL-7-STAT5 axis that facilitates Th1 polarization, IL-21 regulates the differentiation of almost every major subset of CD4^+^ T cells (9). Upon IL-4 engagement, the expression of the Th1 cell master regulator, T-bet, was down-regulated in control CAR-T cells but not in both ICR-expressing T cells. Th2-specific GATA3 expression was up-regulated in control cells while little affected in ICR-expressing T cells. Intriguingly, RORγt expression was dramatically elevated in 4/21 ICR-CAR T cells (Figure 2B). RORγt is the critical transcriptional factor that orchestrates the differentiation of the Th17 cell lineage, whose expression is induced in an IL-21/STAT3-dependent manner (15). To further confirm the Th17 phenotypes of 4/21 ICR-CAR T cells, the expression of CD26, one of the distinguished markers of Th17 cells (16), was assessed and found to be highly expressed in most 4/21 ICR-CAR T cells exposed to IL-4 (Figure 2C). Consistently, CXCR3, the chemokine receptor generally expressed on Th1/Th17 cells (17), was highly expressed on the 4/21 ICR-CAR T cells in the presence of IL-4 (Figure 2D). As expected, 4/21 ICR-CAR T cells secreted higher levels of IL-17A after antigen stimulation, compared with control cells (Supplementary Figure S2). These data support that 4/21 ICR can activate the analogous downstream signaling of native IL-21R and thereby promote Th17-like differentiation in CAR-T cells upon IL-4 engagement.

### 4/21 ICR-CAR T Cells Efficiently Killed Tumor Cells With Attenuated Exhaustion *in vitro*

**Figure 3:**
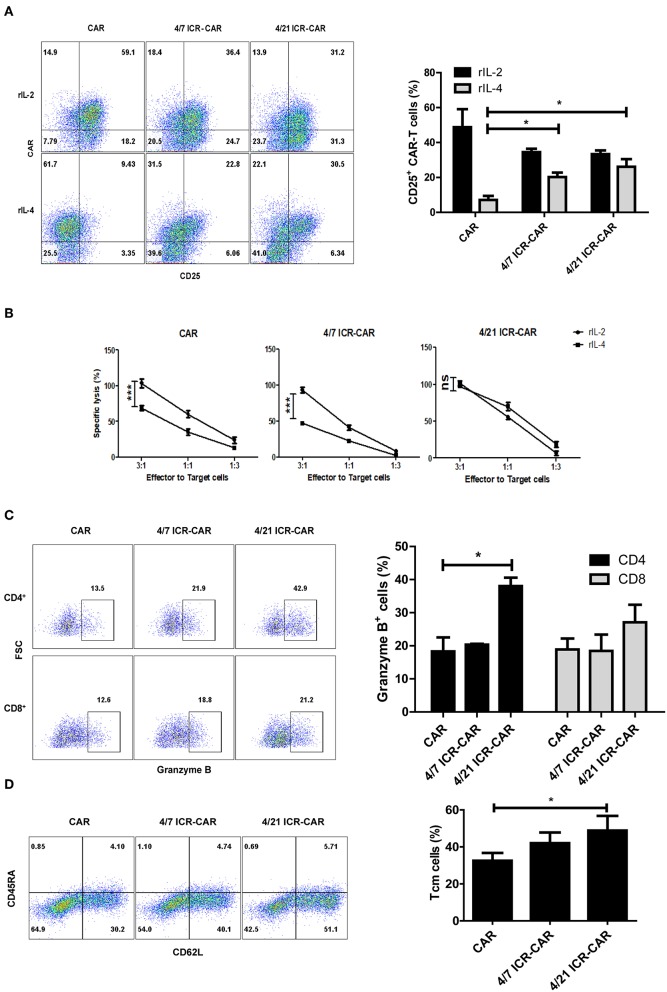
Anti-tumor activities of 4/21 ICR-CAR T cells *in vitro*. **(A)** T cell activation marker, CD25 expression upon IL-4 treatment (20 ng/ml for 48 h) was determined by flow cytometry. **(B)** Following IL-4 exposure, control, 4/7 ICR or 4/21 ICR-CAR T cells were co-incubated with Huh-7 cells at different E:T ratios for 18 h. Cytotoxicity was measured using a standard non-radioactive cytotoxicity assay. **(C)** Granzyme B expression of CAR-T cells was measured by flow cytometry after Huh-7 cell stimulation for 24 h. **(D)** The CD45RA^−^ CD62L^+^ subset of CAR-T cells was detected 3 days after Huh-7 cell stimulation. Representative results from one of three independent experiments are shown. *n* = 3 samples for each group; Graphic results are presented as mean ± SD; ns: not significant; *p < 0.05; ***p < 0.001, one-way ANOVA with Tukey *post-test* for multiple comparison.

The previous study declared that 4/7 ICR-CAR T cells maintained a CD25^+^ activated phenotype in IL-4-rich condition (6). A similar activated phenotype was also observed in 4/21 ICR-CAR T cells (Figure 3A). However, unlike the control and 4/7 ICR-CAR T cells whose cytotoxicity's were significantly compromised by IL-4, 4/21 ICR-CAR T cells retained cytotoxicity in the presence of IL-4 (Figure 3B). Consistent with the data of mRNA quantification, the Granzyme B expression in 4/21 ICR-CAR T cells was higher than in the control and 4/7 ICR-CAR T cells, especially in the CD4^+^ subset, after tumor cell stimulation (Figure 3C). Furthermore, 4/21 ICR-CAR T cells exhibited a larger subset of CD45RA^−^ CD62L^+^ cells (Figure 3D), which is considered to retain the central memory (Tcm) phenotype (18).

**Figure 4:**
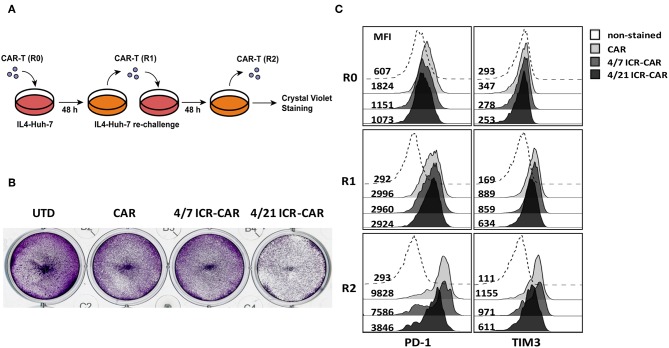
**(A,B)** After 48 h antigen stimulation by IL-4-Huh7 cells (E:T ratio= 1:1), control, 4/7 ICR or 4/21 ICR-CAR T cells were re-challenged with IL-4-Huh7 cells at an E:T ratio of 1:20 for another 48 h. **(C)** T cells were collected for PD-1 and TIM3 expression analysis at indicated time points and tumor cells were stained by crystal violet at the end point. Representative results from one of three independent experiments are shown.

To determine the long-term anti-tumor effects of 4/21 ICR-CAR T cells, we constructed tumor cells producing human IL-4. After being repeatedly challenged with IL4-Huh7 cells, 4/21 ICR-CAR T cells displayed an improved tumor-cell elimination than the control CAR and 4/7 ICR-CAR T cells (Figures 4A,B). Analyses of inhibitory molecule expression revealed that PD-1 and TIM3 expression were minimally induced after two antigen stimulations in 4/21 ICR-CAR T cells, while being greatly upregulated in both the control and 4/7 ICR-CAR T cells (Figure 4C). Thus, our data suggests that in the IL-4^+^ tumor milieu, 4/21 ICR-CAR T cells can maintain an activated but less exhausted status and persistently eliminate tumor cells.

### 4/21 ICR-CAR T Cells Survived and Eradicated Established IL-4^+^ Tumors *in vivo*

**Figure 5:**
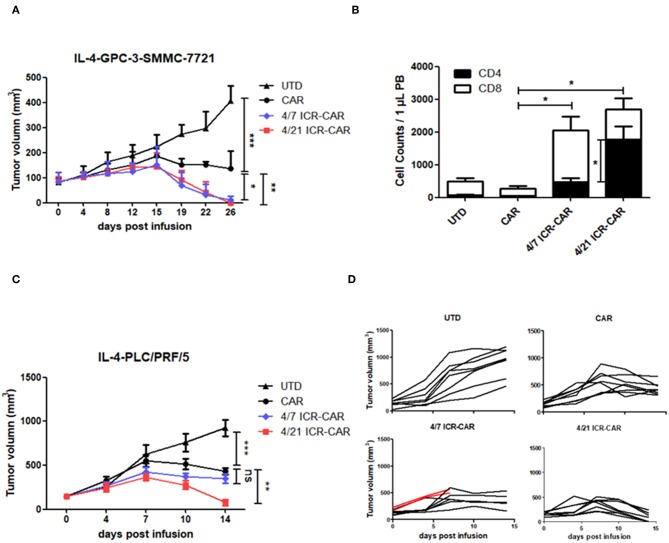
Anti-tumor activities of 4/21 ICR-CAR T cells in established cancer xenografts *in vivo*. **(A)** Mice were subcutaneously inoculated with 3 × 10^6^ IL-4-SMMC-7721 tumor cells on the right flank. 3 × 10^6^ UTD T cells, control, 4/7 ICR or 4/21 ICR-CAR T cells were intravenously injected when tumors had grown to ~100 mm^3^. *n* = 4 mice for each group; **(B)** Peripheral blood samples were collected on 14 days after T cell infusion to determine the T cell survival. T cells were analyzed by anti-CD3/CD4/CD8 antibodies and enumerated. **(C,D)** Mice were subcutaneously inoculated with 3 × 10^6^ IL-4-PLC/PRF/5 tumor cells on the right flank. 3 × 10^6^ UTD T cells, control, 4/7 ICR or 4/21 ICR-CAR T cells were intravenously injected when tumor burden had reached ~200 mm^3^. Red lines in **(D)** indicate the tumor volume of two mice died after T cell infusion. *n* = 8 mice for each group; Data are presented as mean ± SD; ns: not significant; *p < 0.05; **p < 0.01; ***p < 0.001, one-way ANOVA with Tukey *post-test* for multiple comparison.

To investigate the therapeutic effects of 4/21 ICR-CAR T cells *in vivo*, we resorted to a tumor xenograft mouse model. Since Huh-7 tumors had been efficiently cleared by CAR-T cells in our previous animal experiment (11), we adopted two tumor cell lines that were less sensitive to CAR-T cells. In one xenograft model, IL-4-producing SMMC-7721 tumor cells were subcutaneously transplanted into immunodeficient mice. After the tumors had grown to ~100 mm^3^, the mice were administrated CAR-T or untransduced (UTD) control T cells intravenously. In this tumor model, both 4/7 and 4/21 ICR-CAR T cells showed superior anti-tumor effects compared with control CAR-T cells (Figure 5A). A number of CD3^+^ T cells were still detectable in both ICR-CAR T cell-treated mice 2 weeks after infusion, indicating the enhanced persistence of the ICR-CAR T cells *in vivo* (Figure 5B). Interestingly, the proportion of the CD4^+^ subset in the survived T cells was significantly higher in the 4/21 ICR group, which could be explained by the long-lived subset of Th17 cells (19, 20).

In the IL-4-PLC/PRF/5 tumor model, CAR-T cells were challenged with a larger tumor burden (~200 mm^3^). We observed that tumors treated with 4/21 ICR-CAR T cells were rapidly eradicated compared with those in the control group, whereas the tumor clearance was not that efficient by 4/7 ICR-CAR T cells (Figures 5C,D).

## Discussion

4/7 ICR has been invented to reinforce CAR-T cells to withstand the immunosuppressive tumor microenvironment, but whether other combinations of the cytokine receptors could be equally or more effective remains to be explored. Inspired by this, we constructed the novel 4/21 ICR which combines the ectodomain of the IL-4 receptor and endodomain of the IL-21 receptor. 4/21 ICR expressing CAR-T cells achieved enhanced resistance to IL-4, exhibiting the comparable, if not improved, anti-tumor activities to the 4/7 ICR-CAR T cells *in vivo*.

Our findings indicate that 4/7 and 4/21 ICR fundamentally differed in the phospho-STAT signaling cascade. 4/7 ICR activation leads to STAT5 phosphorylation, while 4/21 ICR prefers to activate STAT3 phosphorylation. Importantly, activation of STAT3 is supposed to be one of the critical determinants of CAR-T cell potency. A transcriptomic study has revealed that the genes of STAT3 signatures were enriched in CAR-T cells from complete-responding patients (21). Furthermore, a newly designed CAR with superior anti-tumor capacity has involved the STAT3 signal by integrating the YXXQ motif from IL-21 receptors, which is essential for its optimal functions (22). Therefore, 4/21 ICR would be a new trigger to activate the STAT3 pathway in response to immunosuppressive IL-4, to produce potent antitumor functions in CAR-T cells.

4/21 ICR-CAR T cells presented superior functional activities in our *in vitro* assays. Although IL-4 impaired the cytotoxicity of the control and 4/7 ICR-CAR T cells, 4/21 ICR-CAR T cells retained the cytotoxicity in the presence of IL-4 (Figure 3B). This could be attributed to the increased expression of Granzyme B (Figures 2A, 3C), an important effector molecule that has been reported as a target gene of IL-21 signaling (23). In addition, 4/21 ICR-CAR T cells displayed sustained cytotoxicity with an attenuated exhaustion phenotype in the tumor re-challenge assay. These results could be explained by the Th17-like polarization of 4/21 ICR-CAR T cells, since Th17 cells has been demonstrated to retain a stem cell-like molecular signature with lower expression of exhaustion markers than Th1 cells (20).

Th17 cells have been discovered as a new CD4^+^ effector subset independent of other helper cells. Although CD8^+^ T cells have been recognized as the major effector cells for T-cell cancer immunity, it has become controversial to adopt CD4^+^ or CD8^+^ T cells to generate CAR-T cells. A recent study on glioblastoma-targeted CAR-T cells claimed that CD4^+^ CAR-T cells performed a long-term antitumor response superior to CD8^+^ CAR-T cells (24). In line with this, CD8^+^ CAR-T cells have been demonstrated to be prone to exhaustion and apoptosis upon TCR engagement (25). C. June and his colleagues first explored the efficacy of CD4^+^ ICOS-CAR-T cells generated in the Th17-redirected condition (26). They further raised that the CD26^high^-ICOS-CAR-T cells with Th1/Th17 phenotypes elicited potent tumor elimination and durable persistence, and even outperformed CD8 CAR-T cells in the mesothelioma animal model (27). In the present study, 4/21 ICR-CAR T cells highly expressed RORγt and displayed Th17-like phenotypes *in vitro* and performed potent anti-tumor activities with persistence of CD4^+^ T cells *in vivo*. These results suggest that 4/21 ICR enhances CAR-T cell functions upon IL-4 exposure in a Th17-promoting manner, which is distinct from 4/7 ICR.

As a cytokine driving homeostatic expansion, IL-7 can stimulate T cell proliferation in an antigen-independent manner. A constitutively active form of the IL-7 receptor (C7R) has been engineered for CAR-T cells to improve proliferation, survival and anti-tumor activity (28). Although the investigators did not observe autonomous T cell expansion *in vitro*, the potential safety risk could not be completely obviated. Considering this, the IL-7R mutant has been associated with T-cell acute lymphoblastic leukemia (29). Similarly, given that IL-7 signaling could be over-activated in 4/7 ICR-CAR T cells, if IL-4 is available in abundance outside tumors, it might induce unwarranted expansion. By contrast, IL-21 synergistically induces T cell proliferation with TCR signaling, but has little effect alone (30). Our data also indicates that IL-4 alone did not enhance the expansion of 4/21 ICR-CAR T cells but did in the presence of antigen (Supplementary Figure S3). In our animal experiments, we observed the lethal toxicity of 4/7 ICR-CAR T cells in mice with heavier tumor burdens (Figure 5D, Supplementary Figure S4) early after T cell infusion, while all mice that received 4/21 ICR-CAR T cells survived at the end point of the experiment. The mechanism for 4/7 ICR induced toxicity is currently unclear. Nonetheless, IL-4 levels in patients must be carefully monitored when ICR constructs are clinically translated.

Admittedly, the present research is limited by the immunodeficient mouse model, which lacks the crosstalk between CAR-T cells and the native immune cells. Especially, considering that IL-21 has pleiotropic effects synergizing with various cytokines in T cells, it must be closely scrutinized whether 4/21 ICR could achieve an expected outcome in immune-competent models and clinical trials.

Altogether, in the presence of the inhibitory IL-4 signal, CAR-T cells expressing 4/21 ICR polarized into Th17-like phenotypes and showed potent anti-tumor effects and long-term persistence *in vivo*. Thus, 4/21 ICR is promising to be an alternative design for next-generation CAR-T therapy in IL-4-rich cancers.

## Data Availability

The datasets generated for this study are available on request to the corresponding author.

## Ethics Statement

This study was carried out in accordance with the recommendations of' the Animal Research: Reporting *in vivo* Experiments (ARRIVE) criteria. The protocol was approved by Shanghai Cancer Institute Experimental Animal Care Commission.

## Author Contributions

ZL, YW, and HJ contributed to the conception and design of the study. YW, HL, YS, and RS contributed to data acquisition and statistical analysis. YW wrote the first draft of the manuscript. ZL, HJ, and BS contributed to the manuscript review and editing. All authors approved the submitted version.

### Conflict of Interest Statement

The authors declare that the research was conducted in the absence of any commercial or financial relationships that could be construed as a potential conflict of interest.

## References

[1] Schuster SJ Svoboda J Chong EA Nasta SD Mato AR Anak O. Chimeric antigen receptor T cells in refractory B-cell lymphomas. N Engl J Med. (2017) 377:2545–54. 10.1056/NEJMoa1708566 29226764 PMC5788566

[2] Maude SL Laetsch TW Buechner J Rives S Boyer M Bittencourt H. Tisagenlecleucel in children and young adults with B-cell lymphoblastic leukemia. N Engl J Med. (2018) 378:439–48. 10.1056/NEJMoa1709866 29385370 PMC5996391

[3] Beatty GL Moon EK. Chimeric antigen receptor T cells are vulnerable to immunosuppressive mechanisms present within the tumor microenvironment. Oncoimmunology. (2014) 3:e970027. 10.4161/21624011.2014.970027 25941599 PMC4292547

[4] Anderson KG Stromnes IM Greenberg PD. Obstacles posed by the tumor microenvironment to T cell activity: a case for synergistic therapies. Cancer Cell. (2017) 31:311–25. 10.1016/j.ccell.2017.02.008 28292435 PMC5423788

[5] Leen AM Sukumaran S Watanabe N Mohammed S Keirnan J Yanagisawa R. Reversal of tumor immune inhibition using a chimeric cytokine receptor. Mol Ther. (2014) 22:1211–20. 10.1038/mt.2014.47 24732709 PMC4048899

[6] Mohammed S Sukumaran S Bajgain P Watanabe N Heslop HE Rooney CM. Improving chimeric antigen receptor-modified T cell function by reversing the immunosuppressive tumor microenvironment of pancreatic cancer. Mol Ther. (2017) 25:249–58. 10.1016/j.ymthe.2016.10.016 28129119 PMC5363304

[7] Hinrichs CS Spolski R Paulos CM Gattinoni L Kerstann KW Palmer DC. IL-2 and IL-21 confer opposing differentiation programs to CD8+ T cells for adoptive immunotherapy. Blood. (2008) 111:5326–33. 10.1182/blood-2007-09-113050 18276844 PMC2396726

[8] Markley JC Sadelain M. IL-7 and IL-21 are superior to IL-2 and IL-15 in promoting human T cell-mediated rejection of systemic lymphoma in immunodeficient mice. Blood. (2010) 115:3508–19. 10.1182/blood-2009-09-241398 20190192 PMC2867264

[9] Tian Y Zajac AJ. IL-21 and T cell differentiation: consider the context. Trends Immunol. (2016) 37:557–68. 10.1016/j.it.2016.06.001 27389961 PMC4969098

[10] Wu X Shi B Zhang J Shi Z Di S Fan M. A fusion receptor as a safety switch, detection, and purification biomarker for adoptive transferred T cells. Mol Ther. (2017) 25:2270–9. 10.1016/j.ymthe.2017.06.026 28757080 PMC5628797

[11] Gao H Li K Tu H Pan X Jiang H Shi B. Development of T cells redirected to glypican-3 for the treatment of hepatocellular carcinoma. Clin Cancer Res. (2014) 20:6418–28. 10.1158/1078-0432.CCR-14-1170 25320357

[12] Zeng R Spolski R Casas E Zhu W Levy DE Leonard WJ. The molecular basis of IL-21-mediated proliferation. Blood. (2007) 109:4135–42. 10.1182/blood-2006-10-054973 17234735 PMC1885510

[13] Crotty S Johnston RJ Schoenberger SP. Effectors and memories: Bcl-6 and Blimp-1 in T and B lymphocyte differentiation. Nat Immunol. (2010) 11:114–20. 10.1038/ni.1837 20084069 PMC2864556

[14] Kallies A Xin A Belz GT Nutt SL. Blimp-1 transcription factor is required for the differentiation of effector CD8(+) T cells and memory responses. Immunity. (2009) 31:283–95. 10.1016/j.immuni.2009.06.021 19664942

[15] Korn T Bettelli E Gao W Awasthi A Jager A Strom TB. IL-21 initiates an alternative pathway to induce proinflammatory T(H)17 cells. Nature. (2007) 448:484–7. 10.1038/nature05970 17581588 PMC3805028

[16] Bengsch B Seigel B Flecken T Wolanski J Blum HE Thimme R. Human Th17 cells express high levels of enzymatically active dipeptidylpeptidase IV (CD26). J Immunol. (2012) 188:5438–47. 10.4049/jimmunol.1103801 22539793

[17] Lim HW Lee J Hillsamer P Kim CH. Human Th17 cells share major trafficking receptors with both polarized effector T cells and FOXP3+ regulatory T cells. J Immunol. (2008) 180:122–9. 10.4049/jimmunol.180.1.122 18097011

[18] Gattinoni L Speiser DE Lichterfeld M Bonini C. T memory stem cells in health and disease. Nat Med. (2017) 23:18–27. 10.1038/nm.4241 28060797 PMC6354775

[19] Kryczek I Zhao E Liu Y Wang Y Vatan L Szeliga W. Human TH17 cells are long-lived effector memory cells. Sci Transl Med. (2011) 3:104ra100. 10.1126/scitranslmed.3002949 21998407 PMC3345568

[20] Muranski P Borman ZA Kerkar SP Klebanoff CA Ji Y Sanchez-Perez L. Th17 cells are long lived and retain a stem cell-like molecular signature. Immunity. (2011) 35:972–85. 10.1016/j.immuni.2011.09.019 22177921 PMC3246082

[21] Fraietta JA Lacey SF Orlando EJ Pruteanu-Malinici I Gohil M Lundh S. Determinants of response and resistance to CD19 chimeric antigen receptor (CAR) T cell therapy of chronic lymphocytic leukemia. Nat Med. (2018) 24:563–71. 10.1038/s41591-018-0010-1 29713085 PMC6117613

[22] Kagoya Y Tanaka S Guo T Anczurowski M Wang CH Saso K. A novel chimeric antigen receptor containing a JAK-STAT signaling domain mediates superior antitumor effects. Nat Med. (2018) 24:352–9. 10.1038/nm.4478 29400710 PMC5839992

[23] Zeng R Spolski R Finkelstein SE Oh S Kovanen PE Hinrichs CS. Synergy of IL-21 and IL-15 in regulating CD8+ T cell expansion and function. J Exp Med. (2005) 201:139–48. 10.1084/jem.20041057 15630141 PMC2212766

[24] Wang D Aguilar B Starr R Alizadeh D Brito A Sarkissian A. Glioblastoma-targeted CD4+ CAR T cells mediate superior antitumor activity. JCI Insight. (2018) 3:e99048. 10.1172/jci.insight.99048 29769444 PMC6012522

[25] Yang Y Kohler ME Chien CD Sauter CT Jacoby E Yan C TCR engagement negatively affects CD8 but not CD4 CAR T cell expansion and leukemic clearance. Sci Transl Med. (2017) 9:eaag1209 10.1126/scitranslmed.aag1209 29167392 PMC6944272

[26] Guedan S Chen X Madar A Carpenito C McGettigan SE Frigault MJ. ICOS-based chimeric antigen receptors program bipolar TH17/TH1 cells. Blood. (2014) 124:1070–80. 10.1182/blood-2013-10-535245 24986688 PMC4133482

[27] Bailey SR Nelson MH Majchrzak K Bowers JS Wyatt MM Smith AS Human CD26(high) T cells elicit tumor immunity against multiple malignancies via enhanced migration and persistence. Nat Commun. (2017) 8:1961 10.1038/s41467-017-01867-9 29213079 PMC5719008

[28] Shum T Omer B Tashiro H Kruse RL Wagner DL Parikh K. Constitutive signaling from an engineered IL7 receptor promotes durable tumor elimination by tumor-redirected T cells. Cancer Discov. (2017) 7:1238–47. 10.1158/2159-8290.CD-17-0538 28830878 PMC5669830

[29] Zenatti PP Ribeiro D Li W Zuurbier L Silva MC Paganin M. Oncogenic IL7R gain-of-function mutations in childhood T-cell acute lymphoblastic leukemia. Nat Genet. (2011) 43:932–9. 10.1038/ng.924 21892159 PMC7424552

[30] Parrish-Novak J Dillon SR Nelson A Hammond A Sprecher C Gross JA. Interleukin 21 and its receptor are involved in NK cell expansion and regulation of lymphocyte function. Nature. (2000) 408:57–63. 10.1038/35040504 11081504

