# Supplementary material for: An IL-4/21 Inverted Cytokine Receptor Improving CAR-T Cell Potency in Immunosuppressive Solid-Tumor Microenvironment
Source: Front Immunol. 2019 Jul 19;10:1691. doi: 10.3389/fimmu.2019.01691 (PMC6658891; doi:10.3389/fimmu.2019.01691)
Supplement: Supplementary file 1 [file Data_Sheet_1.docx]

*Supplementary Material*

*
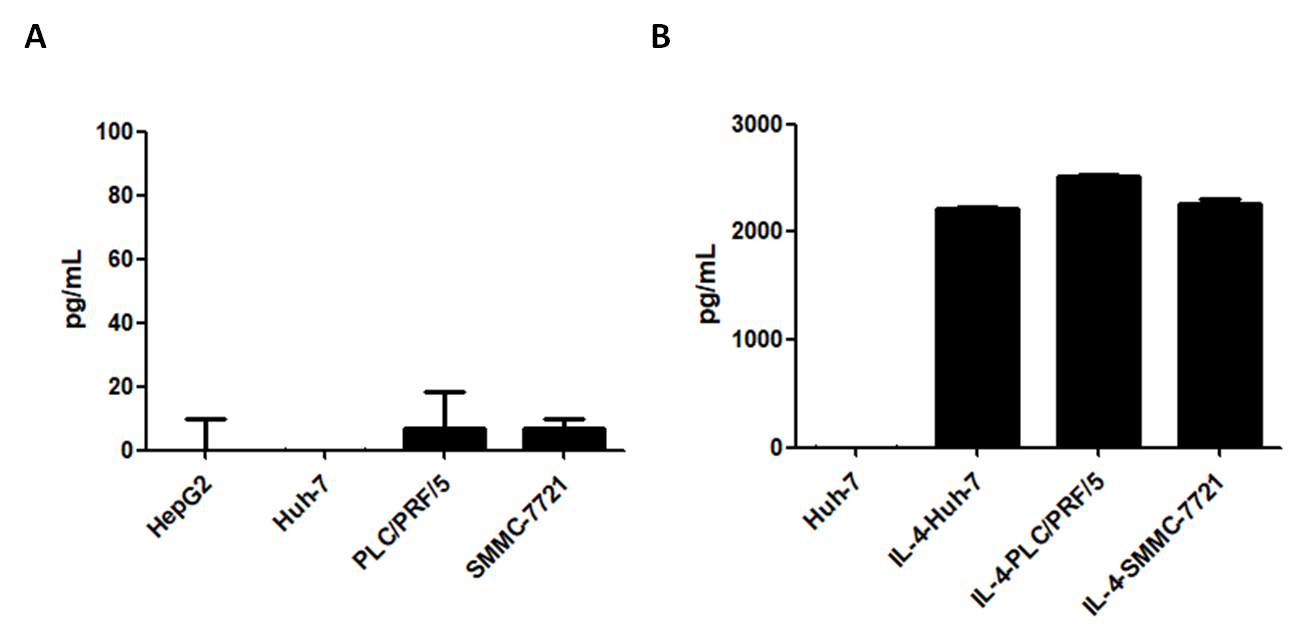
*

**Supplementary Figure S1. IL-4 production of tumor cells used in the study. (A)** Parental and **(B)** IL-4 transduced tumor cells were cultured for 48 h and the supernatant of media was collected for IL-4 measuring via enzyme linked immunosorbent assay (ELISA). n=3 samples for each group; Graphic results are presented as mean±SD.


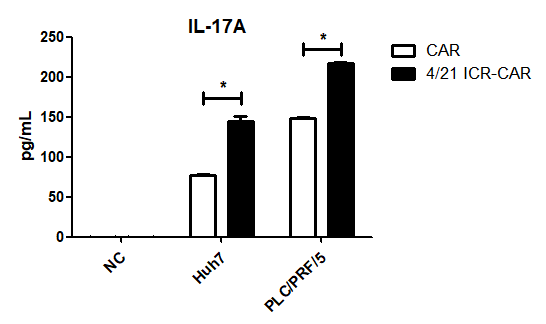


**Supplementary Figure S2. IL-17A secretion of 4/21-ICR CAR T cells upon IL-4 and antigen stimulation.** CAR T cells were pretreated with IL-4 for 48 h, and co-cultured with or without target cells for 24 h. The supernatant of media was collected to measure IL-17A level by ELISA. n=3 samples for each group; Graphic results are presented as mean±SD.


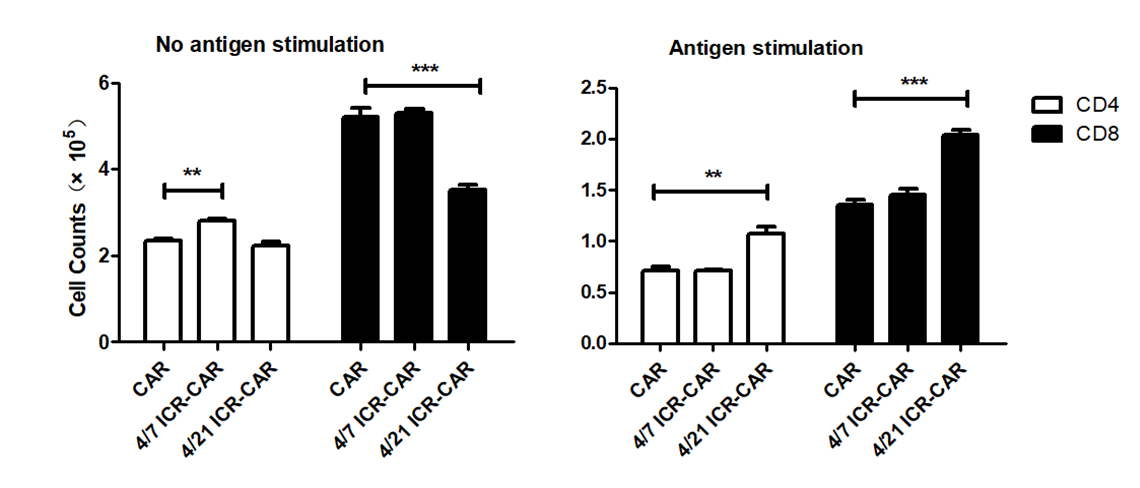


**Supplementary Figure S3. Proliferation of ICR-CAR T cells upon IL-4 treatment with or without antigen stimulation.** CAR T cells were co-cultured with or without Huh-7 cells (E:T ratio = 1:5) upon IL-4 treatment for 96 h. Cells were harvested for flow cytometry analysis. Counting beads (invitrogen, 01-1234) were added for absolute counting. n=3 samples for each group; Graphic results are presented as mean±SD.


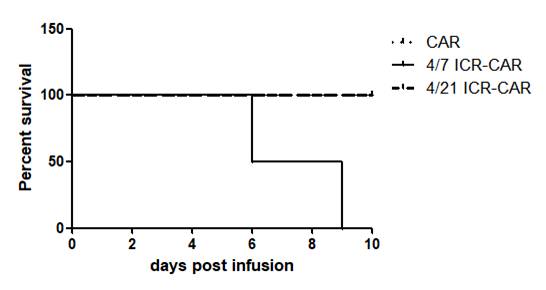


**Supplementary Figure S4 4/7 ICR-CAR T cells showed unexpected lethal toxicity in an IL-4-PLC/PRF/5 xenograft model.** Mice were subcutaneously inoculated with 3×10^6^ IL-4- PLC/PRF/5 cells on the right flank. 3×10^6^ control, 4/7 ICR or 4/21 ICR-CAR T cells were intravenously injected when tumors had grown to approximately 300 mm^3^. n=4 mice for each group.

**Supplementary Table S1. Information of antibodies used in the study**

| **Targets** | **Clone Numbers** | **Catalog Numbers** | **Manufacturers** |
| --- | --- | --- | --- |
| Phospho-STAT3 (Tyr705) | D3A7 | 9145 | Cell Signaling Tech. |
| STAT3 | 124H6 | 9139 | Cell Signaling Tech. |
| Phospho-STAT5 (Tyr694) | C11C5 | 9359 | Cell Signaling Tech |
| STAT5a | 4H1 | 4807 | Cell Signaling Tech. |
| CD3 | SK7 | 11-0036-41 | eBioscience |
| CD4 | RPA-T4 | 562281 | BD |
| CD8 | SK1 | 46-0087-42 | eBioscience |
| CD25 | BC96 | 12-0259-42 | eBioscience |
| CD26 | BA5b | 302709 | BioLegend |
| CXCR3 | 1C6 | 561732 | BD |
| Granzyme B | GB11 | 12-8899-41 | eBioscience |
| CD62L | DREG-56 | 46-0629-41 | eBioscience |
| CD45RA | HI100 | 11-0458-42 | eBioscience |
| PD-1 | eBioJ105 | 46-2799-42 | eBioscience |
| TIM3 | F38-2E2 | 17-3109-42 | eBioscience |

**Supplementary Table S2. Primer sequences used in qPCR**

| **Target genes** | **Forward primers (5’-3’)** | **Reverse primers (5’-3’)** |
| --- | --- | --- |
| BCL6 | CCAGCCACAAGACCGTCCAT | CTCCGCAGGTTTCGCATTT |
| GZMB (Granzyme B) | TGGGGGACCCAGAGATTAAAA | TTTCGTCCATAGGAGACAATGC |
| PRDM1 (Blimp-1) | ACGTGTGGGTACGACCTTG | CTGCCAATCCCTGAAACCT |
| TBX21 (T-bet) | CTGCATATCGTTGAGGTGAA | GTAGGCAGTCACGGCAATGA |
| GATA3 | AGTTGGCCTAAGGTGGTTG | CACGCTGGTAGCTCATACAC |
| RORC (RORγt) | CTGCTGAGAAGGACAGGGAG | AGTTCTGCTGACGGGTGC |
| GAPDH | AACTTTGGCATTGTGGAAGG | GGATGCAGGGATGATGTTCT |
